# Spatial and Temporal Non-Linear Dynamics Analysis and Predictability of Solar Radiation Time Series for La Reunion Island (France)

**DOI:** 10.3390/e20120946

**Published:** 2018-12-08

**Authors:** Miloud Bessafi, Dragutin T. Mihailović, Slavica Malinović-Milićević, Anja Mihailović, Guillaume Jumaux, François Bonnardot, Yannick Fanchette, Jean-Pierre Chabriat

**Affiliations:** 1 Faculty of Sciences and Technology, University of La Réunion, Laboratoire d’Energétique, d’Electronique et Procédés, Sainte-Clotilde, 97715 La Réunion, France; 2 Faculty of Agriculture, University of Novi Sad, 21000 Novi Sad, Serbia; 3 ACIMSI—Center for Meteorology and Environmental Modeling, University of Novi Sad, 21000 Novi Sad, Serbia; 4 Direction Interregionale, 50 Boulevard Chaudron, Sainte-Clotilde 97490, La Reunion

**Keywords:** cumulative daily solar irradiation, La Réunion, tropical island, complexity measures, entropy, chaos, spatial weighted Kolmogorov complexity, Hamming distance, Kolmogorov time predictability mapping

## Abstract

Analysis of daily solar irradiation variability and predictability in space and time is important for energy resources planning, development, and management. The natural intermittency of solar irradiation is mainly triggered by atmospheric turbulent conditions, radiative transfer, optical properties of cloud and aerosol, moisture and atmospheric stability, orographic and thermal forcing, which introduce additional complexity into the phenomenological records. To address this question for daily solar irradiation data recorded during the period 2011–2015, at 32 stations measuring solar irradiance on La Reunion French tropical Indian Ocean Island, we use the tools of non-linear dynamics: the intermittency and chaos analysis, the largest Lyapunov exponent, Sample entropy, the Kolmogorov complexity and its derivatives (Kolmogorov complexity spectrum and its highest value), and spatial weighted Kolmogorov complexity combined with Hamming distance to assess complexity and corresponding predictability. Finally, we have clustered the Kolmogorov time (that quantifies the time span beyond which randomness significantly influences predictability) for daily cumulative solar irradiation for all stations. We show that under the record-breaking 2011–2012 La Nina event and preceding a very strong El-Nino 2015–2016 event, the predictability of daily incident solar energy over La Réunion is affected.

## 1. Introduction

In the last decade, solar energy has become the main source of renewable energy for electrical production. In addition, climate change requires us to adopt new approaches in order to increase our energy autonomy and to improve sustainable development. Currently, the photovoltaic systems help us to achieve these goals in producing low-carbon electrical energy. Technical and energy performances of the photovoltaic panels increased the possibility of using the solar energy, making its exploitation more efficient and economically acceptable. For the decision makers and the companies that install photovoltaic systems, it is of crucial importance to know the available amount of solar energy in a given place and corresponding time duration. The tropical areas are zones with strong potential of solar energy resources where there exist small islands that are currently dependent largely on fossil fuels. For these islands, the prediction of solar resource remains highly important because of the increasing needs for electrical energy and improving sustainable development. Nevertheless, the estimate of the solar resource for one-day time interval remains difficult because of the complexity of unstable weather conditions. Beyond weather-related conditions, complexity is often experienced in the real world. There exist several definitions of complexity, but they are usually related to entropy. Entropy is a measure of disorder. Clausius, Boltzman, and Kolmogorov were among the most famous pioneers on entropy studies [1,2,3]. Boltzmann has introduced the concept of information in his kinetic theory of gases. Shannon [4] has raised the concept of duality between entropy and information in his theory. With the Turing’s machine and theoretical data processing, Kolmogorov drew up algorithmic complexity [5]. He introduced the concept of the minimal message generated with a Turing’s machine [6]. Kolmogorov complexity is an increasingly widespread algorithm that is used in encoding data. Based on this method, Lempel-Ziv [7] developed a widely used universal lossless data compression tool. These original and followed-up algorithms are also used for other scientific purposes in data analysis [8]. To demonstrate the reliability of the application of Kolmogorov complexity measure on the analysis of solar radiation time series, Mihailović et al. [9] showed a complexity decay of solar UV-spectrum (UV-B) received over the period 1990–2007 in Vojvodina region (Serbia). Their study emphasized the practical usefulness of Kolmogorov complexity methodology on the underlying cause of air pollution and climate change impact on observed ground-based UV-B radiation. Recently, Mihailović et al. [10] have focused on solar irradiation predictability, illustrating the importance of the Kolmogorov complexity analysis on daily solar irradiation dataset over La Réunion Island (France) for the period 2013–2015. Several information measures that were derived from Kolmogorov complexity (Kolmogorov complexity spectrum, the maximum Kolmogorov complexity spectrum, and overall Kolmogorov complexity) have been considered in two of the aforementioned studies.

The purpose of this study is (i) to analyse solar radiation over La Réunion with a broader dataset (2011–2015), using the intermittency and chaos analysis and five information measures and (ii) to estimate the solar irradiation predictability over the island during a record-breaking 2011–2012 La Nina event and preceding a very strong El-Nino 2015–2016 event. The estimation of the predictability was quantified by the Kolmogorov time (KT), according to Mihailović et al. [10]. The study is organized as follows. Part 2 describes: (i) the intermittency and chaos analysis, based on the well-known statistical structure function distribution, and the largest Lyapunov exponent; (ii) Sample entropy; (iii) the Kolmogorov complexity and its derivatives (Kolmogorov complexity spectrum and its highest value); and, (iv) spatial weighted Kolmogorov complexity combined with Hamming distance. Part 3 provides information on study locations and pyranometer measurements of daily solar irradiation of 32 stations at La Reunion (France) data for the period 2011–2015. Part 4 includes the results and discussion. Finally, the concluding remarks are given in part 5.

## 2. Materials and Methodology

Because of their intermittent nature, related-weather conditions are often involved in solar irradiation variability at La Réunion [11,12]. To catch the nonlinear and nonstationary behaviour in solar time series, there are numerous methods that have been used for this purpose. Commonly, solar irradiation analysis covers various scientific domains, like a descriptive statistic, the probability for randomness, multifractal, and chaos for deterministic stochasticity and more recently information theory for complexity [10,13,14,15]. Following Mihailović et al. [10], in this study, we briefly describe the Kolmogorov complexity measure and its derivatives applied on daily solar irradiation dataset. For analysis purposes, we also considered two stochastic tools that are usually used for the intermittency and chaos analysis.

### 2.1. Intermittency and Chaotic Process

The intermittency and chaos analysis, presented in this paper, are respectively based on the well-known statistical structure function distribution, and the largest Lyapunov exponent [13,16,17].

Let us assume that we have M time series $x\left(t\right)=\left\{x_{1}\left(t\right),\;x_{2}\left(t\right),\;\ldots ,\;x_{M}\left(t\right)\right\}$, corresponding to measurements of the solar radiation recorded at M stations. Subsequently, we suppose that we have for each station i, a set of N data points $x_{i}\left(t\right)=\left\{x_{i}\left(t_{1}\right),\;x_{i}\left(t_{2}\right),\;\ldots ,\;x_{i}\left(t_{N}\right)\right\}$. The structure function related to the time series $x_{i}\left(t\right)$ is given by $S_{i}{}_{q}\left(\tau \right)={\left|\Delta x_{i}{}_{\tau }\right|}^{q}{}_{t}$ with ${\left|\Delta x_{i}{}_{\tau }\right|}^{q}={\left|x_{i}\left(t+\tau \right)-x_{i}\left(t\right)\right|}^{q}$ and the bracket <>_t_ means the time average. It is clear from the definition of the structure function that $S_{i}{}_{q}$ could be regarded as q-th order moment of the time series $x_{i}\left(t\right)$. For example, 1-st order moment $S_{i}{}_{1}\left(\tau \right)$ can be outlined as $\delta x_{\tau }=S_{i}{}_{1}\left(\tau \right)={\left|\Delta x_{i}{}_{\tau }\right|}_{t}$.

In this study, we seek to see how the 1-st order moment $S_{i}{}_{1}\left(\tau \right)$, 2-nd order moment $S_{i}{}_{2}\left(\tau \right)$, and 4-th order moment $S_{i}{}_{4}\left(\tau \right)$ are staggered in solar radiation time series incremental details giving the information about occurrence of intermittency in them. Now, we draw up the following quantities: (i) the flatness $F\left(\tau \right)=S_{4}\left(\tau \right)/{\left(S_{2}\left(\tau \right)\right)}^{2}$ and (ii) the normalized 1-st order moment $\delta x_{\tau }/\sigma \left(\delta x_{\tau }\right)$, where $\sigma \left(\delta x_{\tau }\right)$ is the standard deviation.

To relate to chaotic analysis, we have used Wolf’s algorithm [18] to compute the Lyapunov exponent spectrum and the largest Lyapunov exponent (LLE). By definition, if $LLE_{i}<1$, then the time series $x_{i}\left(t\right)$ is not chaotic. Conversely, if $0<LLE_{i}<1$ then the time series $x_{i}\left(t\right)$ is considered to have a chaotic pattern.

### 2.2. Sample Entropy and Kolmogorov Complexity

Now, to ensure some clarity in the presentation of sample entropy algorithm, let us consider two subsets $X_{ip}^{m}$ and $X_{iq}^{m}$ extracted from the time series $x_{i}\left(t\right)$. These subsets contain m elements, as follows $X_{ip}^{m}=\left\{x_{i}\left(t_{p}\right),\;x_{i}\left(t_{p+1}\right),\;\ldots ,\;x_{i}\left(t_{p+m}\right)\right\}$ and $X_{iq}^{m}=\left\{x_{i}\left(t_{q}\right),\;x_{i}\left(t_{q+1}\right),\;\ldots ,\;x_{i}\left(t_{q+m}\right)\right\}$ where p≠q. The enable values assigned to p and q are p=1,2,….,N−m and q=1,2,….,N−m. We calculate the sample entropy as $\;SampEn{\left(m,r,N\right)}_{i}=-log\left(\frac{A_{i}}{B_{i}}\right),$ where $A_{i}$ is the number of pair-wise template vector $\left(X_{ip}^{m+1},X_{iq}^{m+1}\right)$ of length m+1 having a distance $d\left(X_{ip}^{m+1},X_{iq}^{m+1}\right)<r$ while $B_{i}$ is the number of pair-wise template vector $\left(X_{ip}^{m},X_{iq}^{m}\right)$ of length m having a distance $d\left(X_{ip}^{m},X_{iq}^{m}\right)<r$. The r is an appropriate threshold value of similarity between the pair-wise template vectors $\left(X_{ip}^{m},X_{iq}^{m}\right)$. Generally, we take the r threshold value or tolerance to be between 0.1 and 0.2 of the standard deviation of the time series $x_{i}\left(t\right)$. The embedding dimension m is usually taken to be 2 [10]. The aforementioned distance $d\left(X_{ip}^{m},X_{iq}^{m}\right)$ is computed using Euclidian metrics. In addition to the definition of $A_{i}$ and $B_{i}$, sample entropy is a positive value that can be 0 for regular and 2.2 or 2.3 for strongly irregular ones.

To assess disorder in solar irradiation time series, except the sample entropy, we use the Kolmogorov complexity and the highest value in the Kolmogorov spectrum algorithms [9]. Let us bear in mind that the mathematical expression of the algorithmic complexity is $K_{T}\left(s\right)=min\left\{\left|p\right|,\;T\left(p\right)=s\right\}$. This formula states that the algorithmic complexity of a string s is shortest program p computed with a Turing’s machine T to gather output s [5,19]. Calculating the algorithmic Kolmogorov complexity $KC_{i}$ of the time series $x_{i}\left(t\right)$ firstly includes encoding the time series with the Lempel-Ziv algorithm [20] into a binarized process and replacing $x_{i}\left(t\right)$ by a new series $s_{i}\left(t\right)$ such as:

(1) $$s_{i}\left(t\right)\{\begin{array}{c} 0\;x_{i}\left(t\right)<x_{i}^{T}\\ 1\;x_{i}\left(t\right)\geq x_{i}^{T} \end{array}$$

where $x_{i}^{T}$ is a threshold value. Usually, the mean of the time series $x_{i}\left(t\right)$ is used as the threshold. Thus, we obtain a set of binary numbers

(2) $$s_{i}\left(t\right)=\left\{s_{i}\left(t_{1}\right),\;s_{i}\left(t_{2}\right),\;\ldots ,\;s_{i}\left(t_{N}\right)\right\}\;\mathrm{with}\;s_{i}\left(t_{p}\right)\{\begin{array}{c} 0\\ 1 \end{array}\;\mathrm{and}\;p=1,2,\ldots ,\;N.$$

Then, we search in the binary time series $s_{i}\left(t\right)$, the overall possible subset sequences that are different from each other. The number of non-matching subsets represents the complexity of the series. Therefore, the value of $C_{i}\left(N\right)$ involved in the binary template $s_{i}\left(t\right)$ is increasingly proportional to randomness. Asymptotically, when the length N of the binary series tends to infinity, the number $C_{i}\left(N\right)$ tends to reach its limit, i.e., $b_{i}\left(N\right)=N/log_{2}N$. Usually, the normalization of the Kolmogorov complexity ${\tilde{KC}}_{i}\left(N\right)$ is given by $C_{i}\left(N\right)=c_{i}\left(N\right)/b_{i}\left(N\right)=c_{i}\left(N\right)log_{2}N/N$. Let us note that Hu et al. [21] derived an analytic expression for $C_{i}\left(N\right)$ in the ${\tilde{KC}}_{i}\left(N\right)$, for regular and random sequences. In addition, they showed that the shorter length of the time series, the larger the $C_{i}\left(N\right)$ value and correspondingly the complexity for a random sequence can be considerably larger than 1.

As mentioned by Mihailović et al. [9], one of the drawbacks of this method is to build up Kolmogorov complexity on searching similarity between sequences without taking into account the amplitude of a time series. To overcome this drawback, he introduced the Kolmogorov complexity spectrum algorithm. The goal of this algorithm is to assess how the computation of the Kolmogorov complexity is sensitive to the threshold value that was used to encode the time series. Basically, we generate a set of binary templates computed iteratively with a set of threshold values defined hereafter. Let us suppose, we split-up the amplitude of the time series $x_{i}\left(t\right)$ into K regular intervals and store the assigned amplitude threshold values $x_{i}^{T_{R}}$. Afterward, we encode the time series $x_{i}\left(t\right)$ for different values of $x_{i}^{T_{L}}$ taken in the threshold set. The encoding time series is now presented by

(3) $$s_{i}^{R}\left(t\right)\{\begin{array}{c} 0\;x_{i}\left(t\right)<x_{i}^{T_{L}}\\ 1\;x_{i}\left(t\right)\geq x_{i}^{T_{L}} \end{array}\;L=1,2,\ldots K_{,}$$

where $x_{i}^{T_{R}}=min\left(x_{i}\left(t\right)\right)+R\left\{\frac{max\left(x_{i}\left(t\right)\right)-min\left(x_{i}\left(t\right)\right)}{K}\right\}$. Thus, the Kolmogorov complexity spectrum is a set of K kolmogorov complexity values ${\tilde{KC}}_{i}{}^{K}\left(N\right)=\left\{{\tilde{KC}}_{i}{}^{1}\left(N\right),\;{\tilde{KC}}_{i}{}^{2}\left(N\right),\;\ldots ,\;{\tilde{KC}}_{i}{}^{K}\left(N\right)\right\}$. Where the value ${\tilde{KC}}_{i}{}^{R_{max}}\left(N\right)=max\left({\tilde{KC}}_{i}{}^{K}\left(N\right)\right)$ with $L_{max}\;\in \left\{1,2,\;\ldots ,\;K\right\}$ being the maximum of the spectrum values. This value can be used to evaluate the discrepancies between the time series average used as threshold to encode the time series and the optimal threshold.

### 2.3. Hamming Distance and Spatial Weighted Kolmogorov Complexity

It is well-known that solar irradiation is strongly related to the weather conditions prevailing at La Réunion [12]. Moreover, Mihailović et al. [10] have pointed out that Kolmogorov complexity is formally a local measure that is spatially independent. They introduced Hamming distance metrics and spatial weighted Kolmogorov complexity to overcome this drawback for mapping. Now, we consider the pair-wise encoded time series $s_{i}\left(t\right)$ and $s_{j}\left(t\right)$ of solar irradiation time series $x_{i}\left(t\right)$ and $x_{j}\left(t\right)$ of two stations i and j, respectively. Hamming distance between this pair-wise binary time series is defined as $d_{ij}{\left(s_{i}\left(t\right),s_{j}\left(t\right)\right)}_{\begin{array}{c} i\neq j\;\\ i,j=1,2,\ldots ,M \end{array}}=\#\left\{p:\;s_{i}\left(t_{p}\right)\neq s_{j}\left(t_{p}\right)\right\}\;,\;p=1,2,\ldots ,\;N$. Formally, we can introduce the XOR logical operator ⊕ then Hamming distance $d_{ij}\left(s_{i}\left(t\right),s_{j}\left(t\right)\right)=\overset{N}{\underset{p=1}{\sum }}s_{i}\left(t_{p}\right)⊕s_{j}\left(t_{p}\right)$. The overall Hamming distance that was assigned to station i is $d_{i}=\overset{M}{\underset{\begin{array}{c} j=1\\ j\neq i \end{array}}{\sum }}d_{ij}\left(s_{i}\left(t\right),s_{j}\left(t\right)\right)$. To steer the spatial homogenization requirement, we have normalized the Hamming distance, as follows ${\tilde{d}}_{i}=\frac{d_{i}}{\max\left\{d_{1},\;d_{2},\;\ldots ,d_{M}\right\}}$. Finally, the spatial weighted Kolmogorov complexity is defined by

(4) $${\tilde{\;KC}}_{i}{}^{H}\left(N\right)={\tilde{d}}_{i}.{\tilde{KC}}_{i}\left(N\right).$$

## 3. Study Locations and Pyranometer Measurements

La Réunion is a small tropical volcanic island (2510 km²) that is located in the Southwest Indian Ocean (21°53′ S, 55°31′ E) and 684 km distant to the east of Madagascar. The area shape of the island is similar to an ellipse roughly oriented along the trade winds direction (South-eastern/North-western axis) and has a rocky peak (Piton des Neiges) that reaches 3000 m height. The local weather is triggered by the mountainous and complex topography with wind valleys, sea/land breeze, and trade winds [11,12]. The temperatures are relatively constant throughout the year with warmer temperature along the coast (23 °C) than inland (15 °C). The air moisture in the island is on average about 60% and sometimes exceeds 90% with tropical cyclones. From day to day, low and overcast clouds are frequent on the slope hill sides and inland [22].

**Figure 1:**
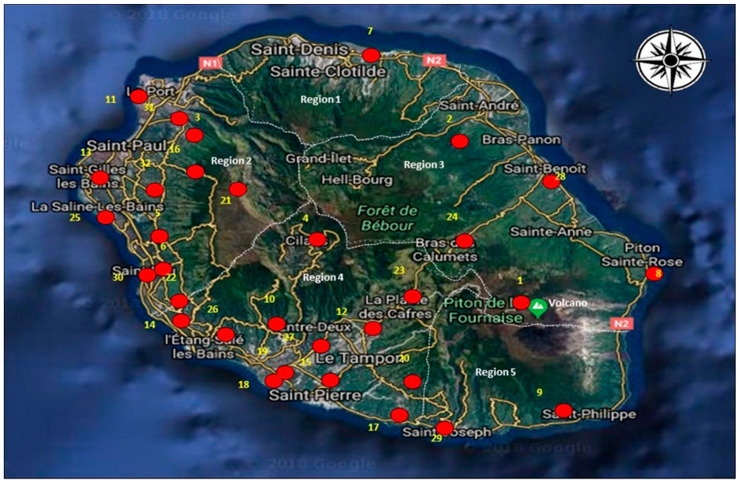
Locations of the 32 radiometric stations on Reunion Island. The boundary of local meteorological regions is indicated with dashed white line [22].

In this study, we use hourly global horizontal solar irradiation from the French Meteorological Service network for the 2011–2015 period [12]. Figure 1 presents the map of the island and displays the 32 located solar irradiation station measurements. The majority of the stations (70%) are located below 500 m. There are six stations that lie between altitudes 500 m and 1000 m and the six remain stations are located beyond 1000m of altitude. Most of the stations are situated on the leeward area of the island (Region 2 and Region 4). There are six pyranometers on the windward side of the island (Region 1 and Region 3). In addition, there are two pyranometers in the south of the island (Region 5), one close to the coast (Saint-Joseph), and the other one near the volcano (Piton de la Fournaise). To summarize, the solar radiation network covers the whole island rather well, although some areas are not covered because of their inaccessibility due to a very steep relief. The overall average pairwise distance between two stations is around 27 km for this network.

**Figure 2:**
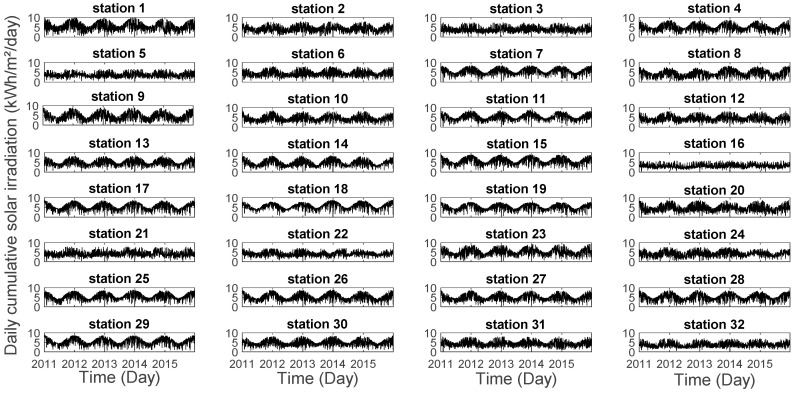
Daily cumulative solar irradiation for the 2011–2015 period recorded for 32 stations at La Réunion.

The total sunshine duration throughout the year is around 3500 h and the global solar irradiation resource is about 1700 kWh/m² per year. As shown in Figure 2, La Réunion Island receives an average amount of incident solar energy of about 5 kWh/m²/day. Moreover, we notice that solar irradiation fluctuations cover a wide time scale from daily to seasonal and interannual range.

**Figure 3:**
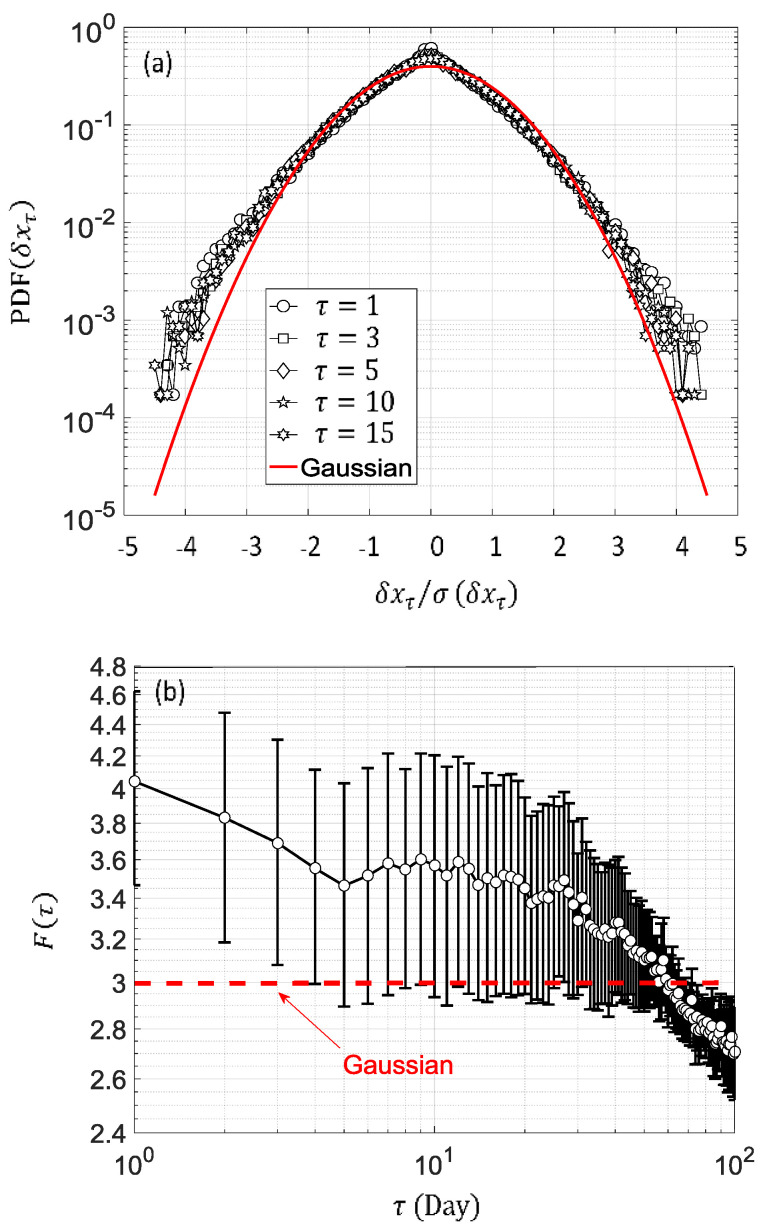
(**a**) Normalized experimental PDFs (Probability Density Function) of the increment $\delta x_{\tau }$ of cumulative daily solar irradiation for time lag τ (1, 3, 5, 10, and 15 days) for the period 2011–2015 record. Red line in Figure 3a is the Gaussian PDFs; (**b**) Flatness F(τ) change distribution with respect to the Gaussian distribution at different time lag τ for the period 2011–2015 record. In Figure 3b, the thick black line connecting empty circles displays the mean of the flatness. Bar error depicts the standard deviation of the flatness computed with 32 stations. The thick dotted red line is the flatness value for Gaussian distribution.

The nature of complexity in time series is related to the way in which fluctuations change with time through broadband time scale [23,24,25]. Basically, the daily solar irradiation fluctuations for each station at La Réunion could be adequately characterized by descriptive statistics to assess a potential complexity footprint in the corresponding time series. Thus, we have used the statistical distribution of increment to measure the temporal behaviour of solar irradiation [13]. A broad increment deviation range from Gaussian distribution within 15 days time interval takes place in daily solar irradiation changes (Figure 3a). In addition, there is a huge dispersion of the flatness around the Gaussian distribution (constant flatness) over a broadband time increment, as shown in Figure 3b. These results reveal some degree of complexity in the time series related to intermittency.

**Figure 4:**
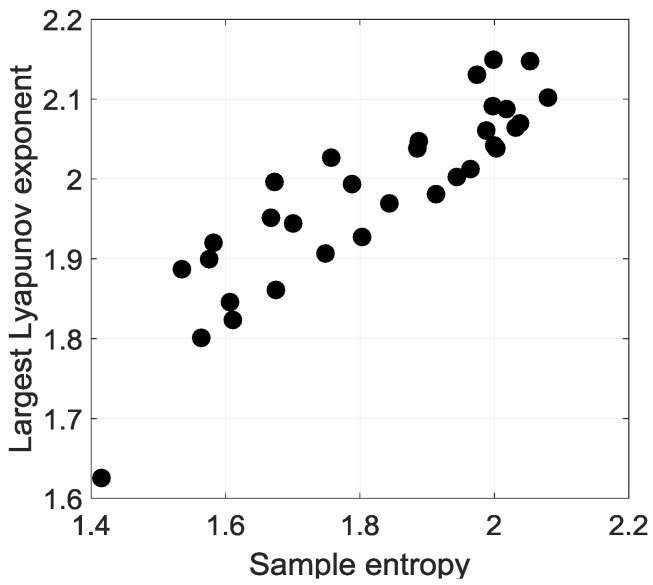
Largest Lyapunov exponent ($LLE_{i}$) versus Sample entropy ($SampEn_{i}$) of the daily cumulative solar irradiation record for the period 2011–2015 (N=1826) days for 32 stations.

As usual, complexity in a solar irradiation time series stands for randomness or disorder. In order to identify the underlying behaviour of these two concepts, there are various methods to study the behaviour of solar irradiation time series [16,23,26]. In the framework of Kolmogorov complexity, we have focused on the need to delineate the predictability of solar irradiation. As it is seen in Figure 4, the daily solar irradiation time series have a sample entropy value greater than 1. As mentioned previously, the function of $SampEn{\left(m,r,N\right)}_{i}$ is the negative of logarithmic. Two similar sequenced of m consecutive data points of a pair-wise template vectors $\left(X_{ip}^{m},X_{iq}^{m}\right)$ remain similar if there are under an appropriate threshold value of similarity r. Thus, we assess that Sample entropy values between 1.4 and 2.2 reveals significant irregularity in the time series. Moreover, largest Lyapunov exponent values placed between 1.6 and 2.2 highlight that the solar irradiation time series at La Réunion contains stochastic chaotic processes (${\mathrm{LLE}}_{i}>1)$. Thus, such Sample entropy and Lyapunov exponent range values are indicators of: (i) very high complexity and (ii) domain of occurrence of stochastic chaos where we cannot use deterministic equations for the forecast. Kolmogorov time gives an estimation of the predictability.

The entropy will have a higher value if the number of sequences in a series is more complicated or without ordered, and vice versa.

**Figure 5:**
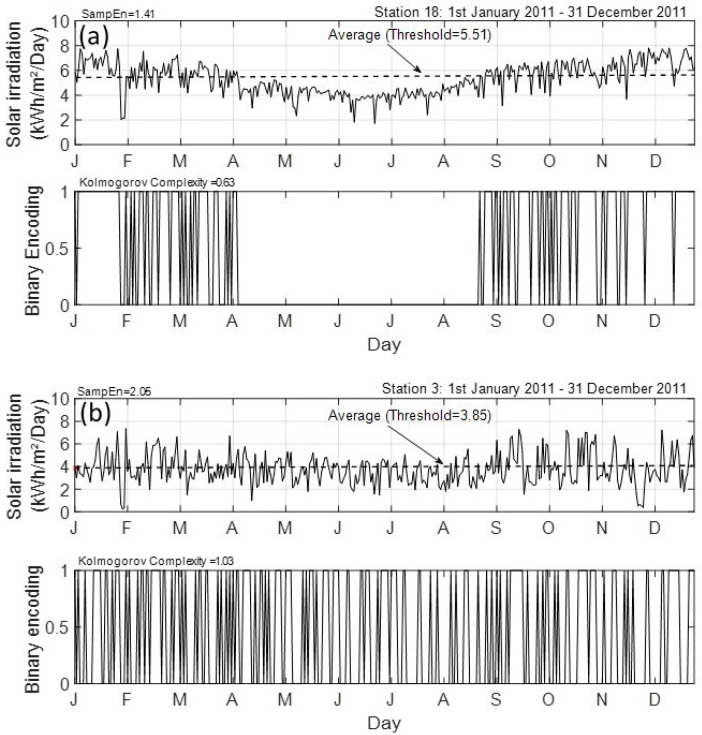
Daily cumulative solar irradiation record and binary encoding for 2011 year for (**a**) station 18 and (**b**) station 3. The thin dashed black line is the mean of the time series used as the threshold amplitude for the binary encoding process. Sample entropy and Kolmogorov complexity are indicated on the left top of each panel.

In further analysis, to quantify solar irradiation predictability at La Réunion, we have followed up the methodology that was proposed by Mihailović et al. [10]. Through two examples, Figure 5 illustrates how it works. To highlight the variations of complexity among 32 stations in the island, we have selected a coastal site (station 18) and an inland site (station 3). For clarity, but without losing generality, we consider only 2011 among the 2011–2015 daily time record periods. To quantify the complexity of real and encoded time series, the computed value of Sample entropy and Kolmogorov complexity are presented in Figure 5. From this figure, it is seen that a higher amount of solar energy is received at the coastal site (5.51 kWh/m²/Day for station 18) than at inland one (3.85 kWh/m²/Day for station 3). Notably, the selected sequences indicate on completely different irregularity and complexity between these two sites. It can be observed that inland time series reveal more irregularity than the coastal time series ($SampEn_{3}=2.05$ against $SampEn_{18}=1.41$) within the assigned complexity state less than the inland site ($KC_{3}=1.03$ against $KC_{18}=0.63$). Referring to the encoding rule, Kolmogorov complexity is directly linked to the departure from the threshold amplitude in binarizing the time series. Here, we use the mean of the time series as the threshold amplitude. During the summer period (November to April), there is more occurrence of threshold amplitude crossing in both time series. Conversely, the pronounced seasonal cycle that was observed on the coastal site leads to assigning a null value to Station 18.

This implies that there is no change in the encoded time series for this station during April to August period. As mentioned by Bessafi et al. [12], this discrepancy throughout the year of the solar irradiation time series between coastal and inland sites is related to the local weather and the overcast clouds prevailing over the island. Despite the difference between these two sites, when complexity is linked to related-weather conditions, the next point that arises is the sensitivity of the Kolmogorov complexity to the threshold amplitude.

**Figure 6:**
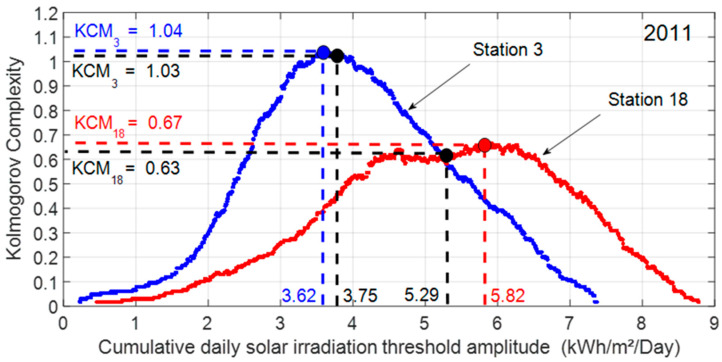
Kolmogorov complexity ${{\tilde{KC}}_{i}\left(N=1826\;\mathrm{days}\right)|}_{i=\left\{3,\;18\right\}}$ and maximum Kolmogorov complexity ${{\tilde{KC}}_{i}{}^{R_{max}}\left(N=1826\;\mathrm{days}\right)|}_{i=\left\{3;\;18\right\}}$ versus cumulative daily solar irradiation threshold amplitude for station 18 and station 3 for year’s 2011.

To overcome this shortcoming, Mihailović and al. [9] introduced the concept of spectral algorithmic complexity. It is an iterative method that generates a set of the threshold values by discretizing the solar irradiation amplitude of the signal. For each threshold value, we encode the time series and provide a set of Kolmogorov complexity values ${\tilde{KC}}_{i}\left(N\right)$. Figure 6 depicts the Kolmogorov complexity spectrum. It shows the distribution of the Kolmogorov complexity ${\tilde{KC}}_{i}\left(N\right)$ versus threshold amplitude used for encoding time series. Usually, the shape of the Kolmogorov spectrum looks like a PDF distribution curve [10]. The value $R_{max}$ defines the threshold value which maximizes the Kolmogorov complexity ${\tilde{KC}}_{i}{}^{R_{max}}$. The sensitivity of the threshold value on the Kolmogorov complexity value is less than 1% with the coastal site and 6% with the inland site.

**Figure 7:**
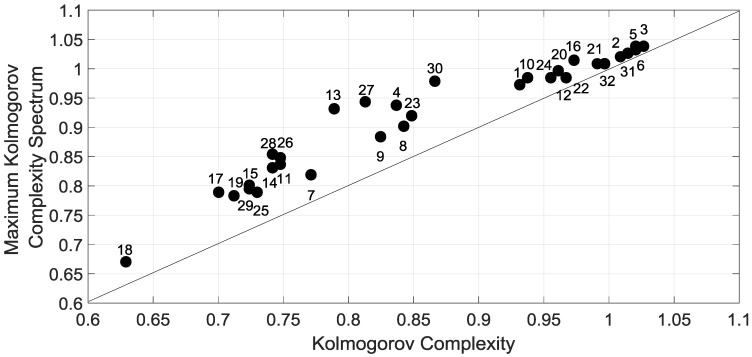
Maximum of Kolmogorov complexity spectrum ${{\tilde{KC}}_{i}{}^{R_{max}}\left(N=1826\;\mathrm{days}\right)|}_{i=\left\{1,2,\ldots ,32\right\}}$ versus Kolmogorov complexity ${{\tilde{KC}}_{i}\left(N=1826\;\mathrm{days}\right)|}_{i=\left\{1,2,\ldots ,32\right\}}$ of 32 stations for the period 2011–2015.

In the extension of this analysis, we have compared the maximum Kolmogorov complexity ${\tilde{KC}}_{i}{}^{R_{max}}\left(N\right)$ obtained with the spectral method and the Kolmogorov complexity ${\tilde{KC}}_{i}\left(N\right)$ obtained with the mean used as the threshold amplitude for the overall sites. Figure 7 emphasized the threshold optimization with Kolmogorov complexity values ${\tilde{KC}}_{i}\left(N\right)$. The results concerning the means are similar as with the spectrum method. The difference between the two methods is less than 1% for inland sites and around 5% for coastal sites. Hereafter, we propose to use the mean as a threshold amplitude to encode the time series.

**Figure 8:**
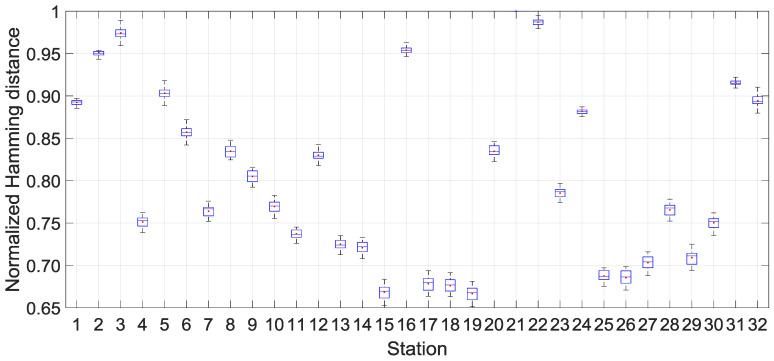
Dissimilarity between the pair-wise stations among 32 stations for the 2011–2015 period. Boxplot depicts the statistics of the Normalized Hamming distance ${{\tilde{d}}_{i}=\frac{d_{i}}{\max\left\{d_{1},\;d_{2},\;\ldots ,d_{32}\right\}}|}_{i=\left\{1,\;2,\;\ldots ,32\right\}}$ for each station. Station 21 does not have boxplot associated as it is used to normalized the Hamming distance between pair-wise stations.

In previous analysis, the Kolmogorov complexity algorithm is built on a standalone solar irradiation time series. The result corresponding to the Kolmogorov complexity computed independently to the surrounding complexity is then a local measure of complexity. It is clear that solar irradiation is spatially dependent over the island at various spatial scale [15]. To combine both the local and broader spatial scale, we have extended the local Kolmogorov complexity ${\tilde{KC}}_{i}\left(N\right)$ to a spatial weighted Kolmogorov complexity ${\tilde{KC}}_{i}{}^{H}\left(N\right)$ using pair-wise dissimilarity between binarized time series. The distance metric that was used in this study to measure dissimilarity is the Hamming distance [10]. Though the solar irradiation network used in this study is moderately dense, we have continued to a test of the robustness of the spatially weighted method. For each site, we have withdrawn one site to proceed to the leave-one-out method. Thus, we hold a statistic of the overall pair-wise similarity value for each station and it is presented in Figure 8. The normalized dissimilarity between daily solar irradiation time series recorded at La Réunion during the 2011–2015 is relatively high, as it varies between 0.66 and 1. Station 21 reaches the highest dissimilarity with the remaining station. This is a particular inland site that is located in an area with the persistent overcast cloud through the year [12]. In accordance with the boxplot size, there is a weak dispersion of the Hamming distance for each site. On the other hand, the dissimilarity measure with Hamming distance is confident through leave-one-out requirement and ensures some robustness on our spatial weighted Kolmogorov complexity methodology.

**Figure 9:**
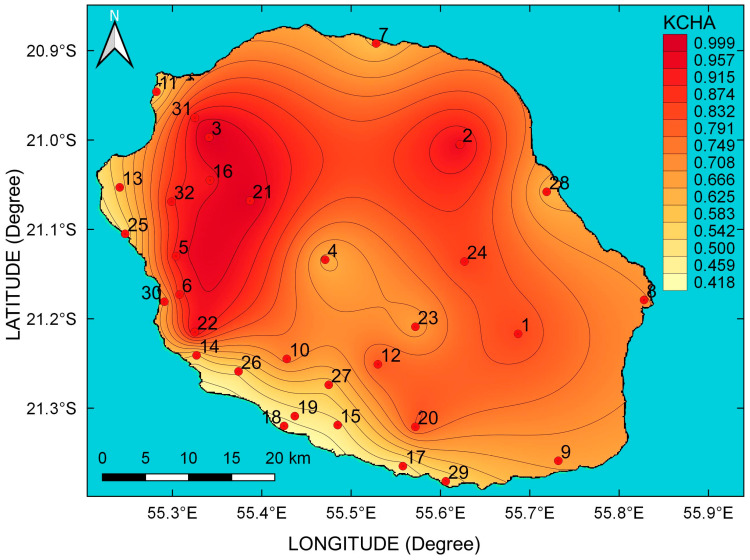
Distribution of the spatial weighted Kolmogorov complexity measure ${{\tilde{KC}}_{i}{}^{H}\left(N=1826\;\mathrm{days}\right)|}_{i=\left\{1,2,\ldots ,32\right\}}$ of cumulative daily solar irradiation of 32 stations for the 2011–2015 period.

Thorough attention to spatial weighted Kolmogorov complexity mapping of daily solar irradiation at la Réunion has been devoted in a recent study [10]. In this previous paper, the mapping has been carried out with a set of 11 pyranometers that covers the 2012–2013 period of the time record. Figure 9 displays spatial weighted Kolmogorov complexity mapping achieved with 32 pyranometers. Moreover, we have used a solar irradiation time series that spans over a longer period from 2011 to 2015. Roughly, the results are according to the previous study. We can retrieve more efficiently and with greater detail the spatial solar radiation complexity in the island. In addition, we notice that the north-west slope hill exhibits the highest complexity. As mentioned previously, this region is an overcast area. Conversely, the south and west coast experience daily solar irradiation with the lowest complexity. Finally, the eastern coast, the inland cirques, and volcano present an intermediate level of complexity. Thus, there is an east/west contrast with well-pronounced complexity northwest slope hill side.

**Figure 10:**
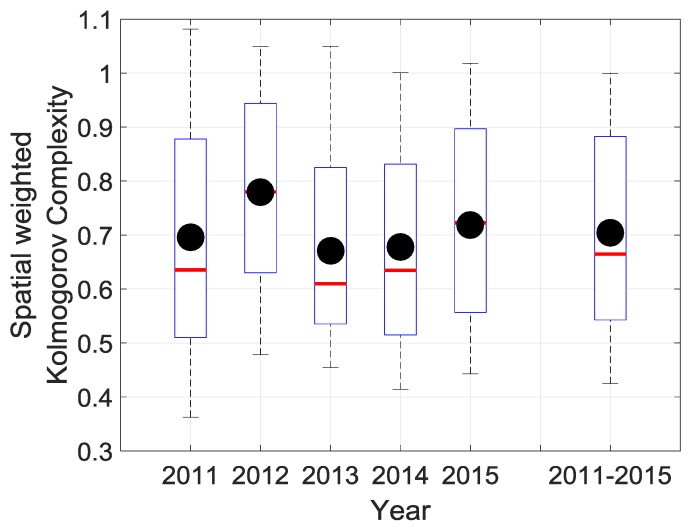
Statistics of the spatial weighted Kolmogorov complexity measure ${{\tilde{KC}}_{i}{}^{H}\left(N\right)|}_{i=\left\{1,2,\ldots ,32\right\}}$ of cumulative daily solar irradiation of 32 stations for each year of the 2011–2015 period. For each year, spatial weighted Kolmogorov complexity is computed with N=365 days. The statistics of 2011–2015 (boxplot on the right side of the graph) correspond to the whole five years and spatial weighted Kolmogorov complexity is computed with N=1826 days.

At this point, in the paper, algorithmic complexity analysis has been conducted while using the entirety of the time series. Subsequently, it is clear that those results of encoding data are dependent on the aforementioned length of the time series. Thus, the complexity measure could be of particular interest to study solar irradiation predictability under shorter time frames within the time series. Firstly, we have focused our interest on complexity measure over the one-year timeframe to check how the complexity of daily solar irradiation at La Réunion behaves, on a yearly basis over the 2011–2015 period. Figure 10 shows meaningful features regarding the evolution and variability of the complexity over the island from 2011 to 2015. On average complexity and its variability fluctuate significantly from one year to another, reflecting some disturbances on incident solar energy received over the island. Consequently, we notice that 2011 was the year with the largest variability range of Kolmogorov complexity value. In contrast, 2013 was the year with the lowest complexity and interquartile range. The complexity range for 2011 was about (0.36–1.08) and a median value of 0.63 in comparison to (0.45–1.04) and the median value of 0.60 in 2013. Moreover, between these two years, 2012 was the year with the highest complexity.

Such noticeable yearly complexity changes could be related to a seventh breaking-record of the 2011–2012 La-Nina event in the Pacific. This event has been reported to have had a huge impact on climate to the extent that it was felt as far as Australia and in the Indian Ocean [27,28]. The La Nina event preceded the very strong 2015–2016 El Nino event. This climate disturbance can impact the Walker circulation in the Pacific and the Indian Ocean, which can lead to rainfall extremes in Australia, Africa, Madagascar, and the islands of Mascareignes (La Réunion, Mauritius, Rodrigues,…) [29,30]. The 2013–2014 period is a neutral ENSO period. We note that large-scale disturbances can interfere with the local weather and affect indirectly the incident solar energy at La Réunion. During the 2011–2012 La Nina event, we noticed that La Réunion received a daily average of 4.55 kWh/m² of solar irradiation in contrast to the climatological mean of 4.63 kWh/m² corresponding to a decrease of 1.7% of incident solar energy. However, following that event, the daily average of solar irradiation was 1.2% greater than the climatological mean with an amount of 4.68 kWh/m² for the neutral period. In 2015, the incident solar energy is 1.5% less than the climatological mean with a daily average of 4.56 kWh/m². This sequence of negative–positive–negative departure from the climatological mean during the 2011–2015 period reveals that interannual event has a direct impact on the solar budget of the island. This could be related to a modulation of cloud cover activities during the ENSO oscillation. As shown by Bonnardot et al. [31], during the 2011–2012 period, the daily insolation duration is 6h50′, which corresponds to a decrease of 3.1% in comparison to the climatological mean of insolation duration of 7h03′. However, following that period, daily insolation duration was 4.1% greater than climatological mean with an insolation duration of 7h21′ during the neutral period. In 2015, the insolation duration is about 6h58′, corresponding to a decrease of 1.2% in contrast to the climatological mean of insolation duration [31].

Consequently, both daily solar irradiation complexity and predictability seem to be influenced by local cloud cover activity as well as modulated by the interannual ENSO event.

**Figure 11:**
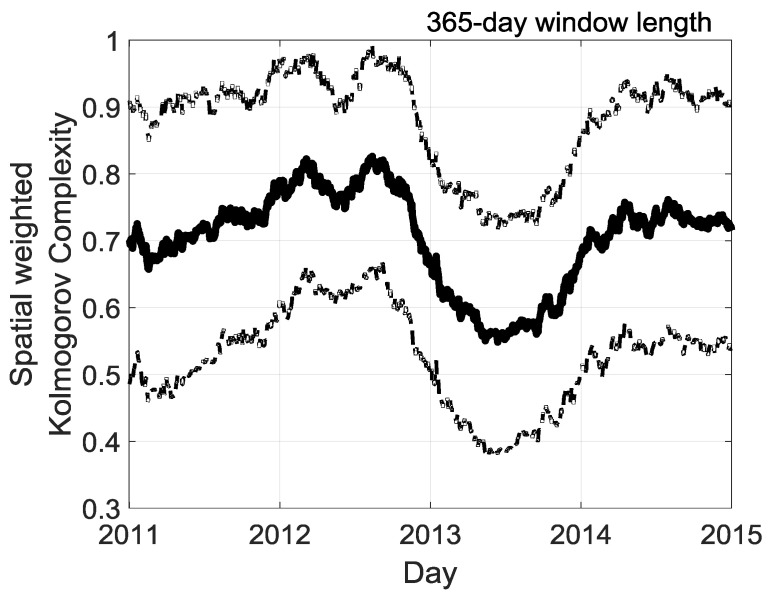
365-days size of moving window of the spatial weighted Kolmogorov complexity measure ${{\tilde{KC}}_{i}{}^{H}\left(N=1826\;\mathrm{days}\right)|}_{i=\left\{1,2,\ldots ,32\right\}}$ of cumulative daily solar irradiation of 32 stations for each year of the 2011–2015 period. The tick solid black line is the average of the 32 stations. Thin dotted black line depicts the standard deviations of the 32 stations. For each day an assigned value of average and standard deviation of ${{\tilde{KC}}_{i}{}^{H}\left(N=1826\;\mathrm{days}\right)|}_{i=\left\{1,2,\ldots ,32\right\}}$ are computed using a centered window with a length of 365 days.

The local weather prevailing over the island on a seasonal scale is a complex mixture of meteorological disturbances extending over a broadband scale of time (ranging from day to year) and space (ranging from meters to hundred kilometers). In addition, this space and time pattern can be modulated from year-to-year by interannual oscillation. Therefore, we try to catch the evolution of the complexity over La Réunion. Figure 11 illustrates the daily evolution of solar irradiation complexity with a focus on the moving window of one-year size. We recognize again the persistent and significant interannual modulation with the highest complexity in 2012 and the lowest in 2013.

**Figure 12:**
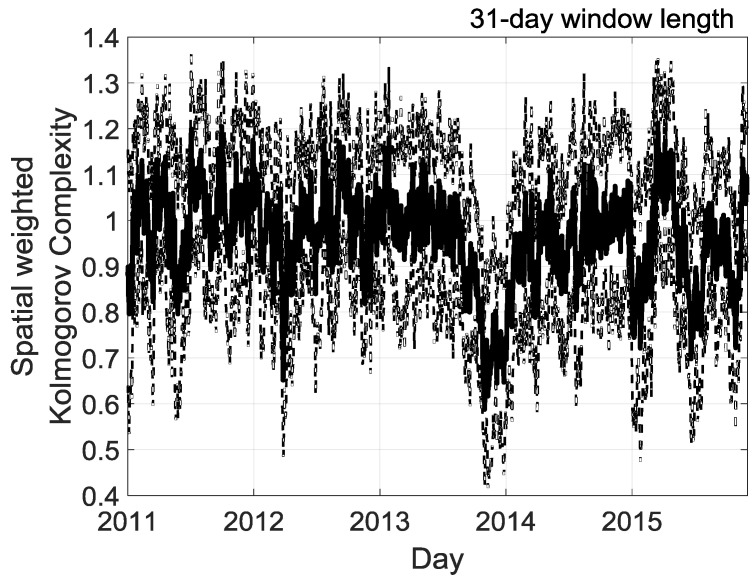
31-days size of moving window the spatial weighted Kolmogorov complexity measure ${{\tilde{KC}}_{i}{}^{H}\left(N=1826\;\mathrm{days}\right)|}_{i=\left\{1,2,\ldots ,32\right\}}$ of cumulative daily solar irradiation of 32 stations for each year of the 2011–2015 period. The tick solid black line is the average of the 32 stations. The thin dotted black line depicts the standard deviations of the 32 stations. For each day an assigned value of average and standard deviation of ${{\tilde{KC}}_{i}{}^{H}\left(N=1826\;\mathrm{days}\right)|}_{i=\left\{1,2,\ldots ,32\right\}}$ is computed using a centered window with a length of 31 days.

We extend the analysis with a one-month window size to capture high-frequency changes in complexity (Figure 12). With a 31-day size moving window, the year 2013 again seems to be a particular year but with a considerable drop in complexity by the end of the year 2013. Moreover, further details are observed throughout the year with high complexity fluctuations in summer (November to April). On average, the 31-day size moving window gives higher complexity than if the 365-days size moving window was used. We obtained a minimum correlation of −0.40 at lag −2 months between La Nina event (2011–2012 period) and solar irradiation complexity at La Réunion. The maximum lag correlation of +0.40 between Neutral ENSO (2013) and Kolmogorov complexity at La Réunion is at −3 months. This cross-correlation analysis reveals a time delayed correlation between the 2011–2015 ENSO index time series and Kolmogorov complexity times series at La Réunion.

**Figure 13:**
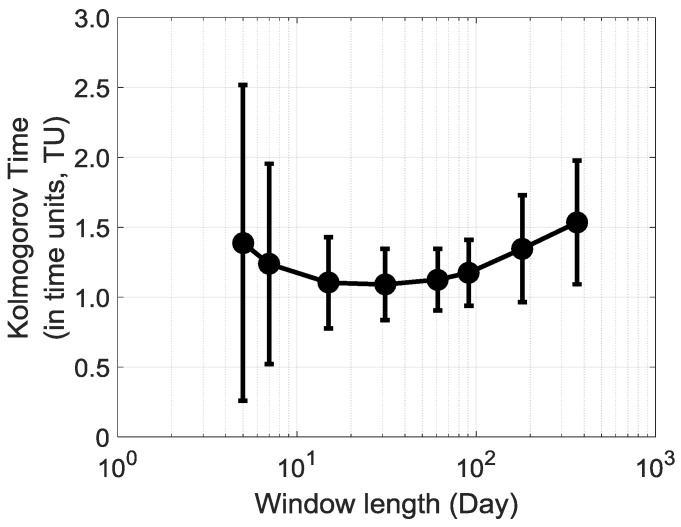
The Values of Kolmogorov time (KT) in time units (TU) for daily cumulative solar irradiation time series of 32 stations at La Reunion for 2011–2015 period. The tick black circles display the Kolmogorov time average of 32 stations. Error bar depicts one standard deviation of the Kolmogorov time.

Based on the previous analysis of a moving window, the next point that arises is the influence of window size on the predictability of daily solar irradiation at La Réunion. In this study, we have used Kolmogorov time (inverse to Kolmogorov complexity in time unit (TU)), as defined by Mihailović et al. [10]. Figure 13 is a first response to the above question displayed on the semi-logarithmic graph. In our study, we have limited our analysis to the size of moving window ranging from five days to one year. We find that Kolmogorov time does not change linearly with window size. The curve is a parabola, with a minimum of 1.1 unit of time being obtained roughly for one month window length. Beyond and below this minimum, Kolmogorov time and its variability increase. The highest Kolmogorov time variability is obtained below 10 days and it could reflect the limitation of using the mean of the time series to compute the threshold amplitude. In addition, the coefficient of variation of the Kolmogorov time is relatively constant above 10-days window size with an average of 25%. This highlights a moderate dispersion of the Kolmogorov time within the 10–365 time range.

It is indicative that solar irradiation time series exhibit a modulation of complexity within the intra seasonal time scale. The predictability seems to be reduced for window size between two weeks and two months. The interquartile range of time predictability for this window size range is in average about 0.3 TU, whereas it is 0.5 TU for the remaining window size. Solar irradiation is mainly driven by the cloud cover activity, which is triggered by local thermodynamic (instability, moist air) and dynamical processes (trade wind, katabatic/anabatic wind, orographic wind) [10]. Tropical storms and cyclones, intraseasonal tropical oscillation, like MJO (Madden Julian Oscillation) and Indian Ocean Dipole (IOD), and interannual oscillations (ENSO events) are superimposed on synoptic scale perturbations [10,11,22,31].

Consequently, if we look into the 2011–2015 solar irradiation time series with a window size between two weeks and two months, then we can catch the footprint of almost all intraseasonal, seasonal and interannual perturbations and explain the decrease of predictability with those window sizes.

**Figure 14:**
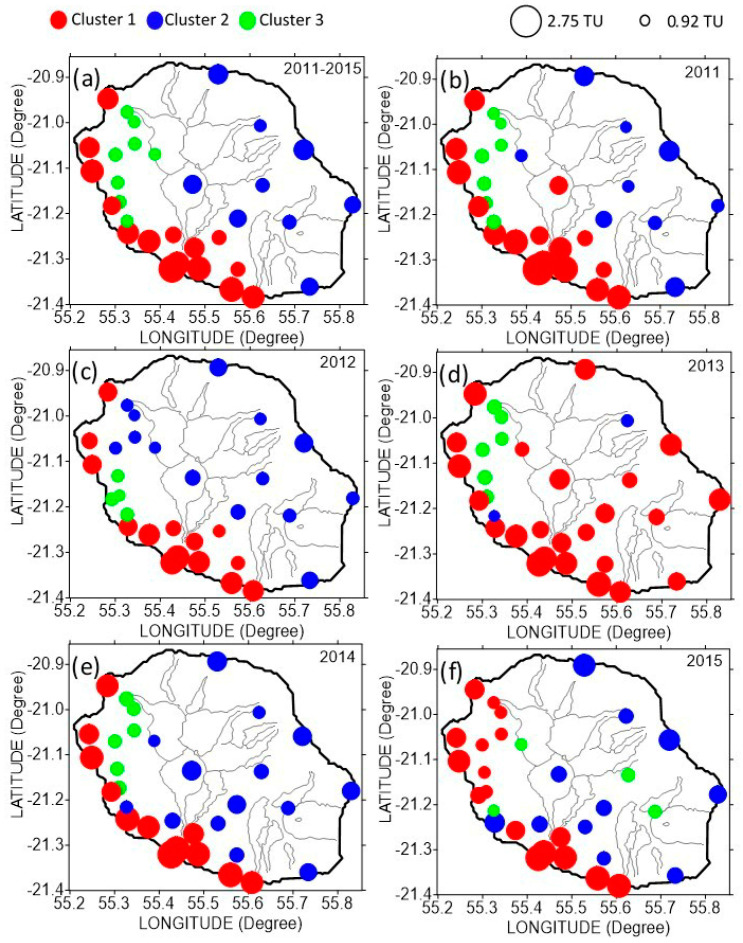
Clustering of Kolmogorov time (KT) for daily cumulative solar irradiation time series of 32 stations at La Reunion for (**a**) the 2011–2015 period, and for year (**b**) 2011, (**c**) 2012, (**d**) 2013, (**e**) 2014, and (**f**) 2015. On the top left of the figure, the coloured filled circle represents the three different class for clustering. On the top right of the figure, the two black circles denote the minimum and maximum Kolmogorov time in time unit (TU).

To highlight the year by year evolution of the spatial pattern of solar irradiation complexity and predictability, we have focused on the joint use of spatial weighted Kolmogorov complexity similarity and Kolmogorov time. We have undertaken a cluster processing that groups the solar station according to similar complexity. We have retained three groups in the clustering process and for each labelled station we have assigned an individual corresponding Kolmogorov time. Moreover, the clustering has been achieved using an unsupervised method of ascending hierarchical classification on the hamming distance set. In summary, Figure 14 shows the geographical clustering of complexity and the Kolmogorov time results for the 2011–2015 period and the individual years from 2011 to 2015. Globally, there is a clear geographical partition about solar complexity with one group that is located along the south and west coast, the other along the west and inland, except for the northwest slope hill that formed the last group. We notice that solar irradiation observed during 2013 reveals a more homogeneous complexity and predictability in contrast to 2012 during which the Kolmogorov time is lower. In addition, the predictability is of the same order along the coast parallel to the southeast-northwest direction than inland and lowest over the overcast northwest slope hill area.

## 4. Conclusions

In this work, we analyse the complexity and predictability of the daily solar irradiation recorded with 32 stations for the period 2011–2015 in Réunion island. To outline the nature of complexity in time series, we have computed the sample entropy and the highest Lyapunov exponent. The result reveals that fluctuations in daily solar irradiation are highly irregular and time series with an occurrence of stochastic chaos. Thus, to address the issue of predictability with stochastic time series, we conduct the analysis with Kolmogorov complexity and its derivatives. Firstly, the encoding solar data into binary time series was done using the mean value of time series elements as a consistent threshold value. For each station, we demonstrated that the difference between the optimum threshold value that was retrieved with the Kolmogorov spectrum and the mean value of time series elements is relatively small. Following the study by Mihailović et al. [10], we have performed a spatial weighted complexity with Hamming distance to encompass the regional scale in the measure of the level of complexity. The spatial stability of this metric has been assessed with a leave-one-out test. Subsequently, we draw-up a map of the weighted Kolmogorov complexity of daily solar irradiation with a much denser radiometric network. The result highlights more details about the spatial pattern of complexity, which is the most interesting in the analysis of daily solar irradiation complexity in the island. As pointed out by Mihailovic et al. [10], complexity is a measure of randomness. He showed that solar irradiation complexity conceptually reflects the number of occurrences of positive-negative or negative-positive deviation of the daily incident solar energy from the threshold. In the real world, solar irradiation that is received on ground is mainly related to the cloud cover activity in the lower troposphere within the boundary layer and the troposphere. The cloud formation and movement over the island are often the result of a complex interactions between thermodynamic (instability, moisture) and dynamic (Venturi effect, katabatic/anabatic, and orographic wind) processes [11]. The northwest slope hill is an overcast area with highly variable weather and cloud cover. We found in the time series a large number of occurrences of positive-negative or negative-positive deviation from the mean, which explains the high complexity value and intermittency in this area. Conversely, lower randomness of daily solar irradiation time series prevails along the eastern and western coasts. Parallel to the southeast-northwest direction, there is an acceleration of the trade winds, which dynamically increases vertical stratification, stability of the air, and thus inhibits low level cloud development. Moderate complexity along the eastern coast (leeward) and inland suggests an intermediate competition between thermodynamics and dynamic processes.

To adequately assess the annual evolution of Kolmogorov complexity over the period 2011–2015, we studied the level of complexity for each year separately. We can highlight an interannual modulation of randomness of daily solar irradiation time series at La Réunion. There is a qualitative correlation between ENSO and complexity during this period. The interannual modulation of complexity seems to be related to breaking-record ENSO events (2011–2012 La-Nina and 2015–2016 El-Nino). There is a significant increase of randomness during the 2011–2012 period, whereas the randomness is lower during the 2013 year. The highest variability of complexity is encountered during the 2011 year. This modulation is also retrieved using the running complexity with the moving window having the size of 365 and 31 days, respectively. In addition, we showed that the Kolmogorov time, which is an indicator of the predictability, varies in dependence to the moving window size in calculating the running complexity used in the analysis. There is a high dispersion of the Kolmogorov time with the variation of window size, which is lower than 10 days. Beyond this window size, we notice that predictability increases and the coefficient of variation of the Kolmogorov time is rather constant around, i.e., 25%. Clustering analyses were conducted and we have noticed that the predictability evolved between 2011 and 2015, confirming the aforementioned results about the spatial pattern that was obtained with Kolmogorov. Nevertheless, it seems that interannual modulation by ENSO events, which has a world impact, significantly alter the spatial pattern of the predictability in the island during the period 2011–2012 La-Nina and 2015–2016 El-Nino episodes). Nevertheless, the global spatial pattern of complexity is roughly maintained through the 2011–2015 period. The competition between thermodynamics and dynamics prevailing over the island is modulated by the large-scale condition, which triggers the solar variability through local cloud cover activity. This work shows that spatial weighted Kolmogorov complexity and Kolmogorov time provide significant insights into the predictability of daily solar irradiation for a small tropical island.
